# Supplementary material for: Avoiding siloed approaches: Integrating psychological insights into sustainable farming
Source: PLoS One. 2024 Oct 14;19(10):e0301881. doi: 10.1371/journal.pone.0301881 (PMC11472944; doi:10.1371/journal.pone.0301881)
Supplement: S1 Table — (DOCX) [file pone.0301881.s001.docx]

Supporting Information

**S1 Table Exploratory Factor Analysis using Maximum Likelihood Extraction and Promax Rotation of the NetZero farming items.**

| Factor 1: Sustainable Agricultural Commitment and Stewardship | | | | | | | |
| --- | --- | --- | --- | --- | --- | --- | --- |
| I would find personal satisfaction and enjoyment in achieving emission reduction targets through my farming practices. | .794 | .085 | .005 | -.130 | -.149 | .048 | .045 |
| Working towards emission reduction targets is in harmony with my values and beliefs about sustainable farming | .764 | .179 | -.014 | -.087 | -.095 | -.051 | -.006 |
| I have a positive attitude towards integrating emission reduction targets into my farming practices | .733 | .177 | -.094 | -.033 | -.194 | .046 | .012 |
| Striving to meet emission reduction targets contributes to my growth as a farmer and a steward of the land | .715 | .141 | .035 | -.090 | .043 | .032 | -.037 |
| I am actively open to adopting new methods and technologies that help achieve emission reduction targets | .679 | .145 | -.001 | .001 | -.153 | -.071 | .122 |
| I find personal satisfaction in implementing practices that increase my farm’s resilience to climate change | .660 | -.110 | .008 | .373 | -.182 | .091 | .051 |
| I have a positive attitude towards adopting practices that increase adaptation and resilience to climate change in agriculture | .660 | -.107 | -.034 | .345 | -.191 | .091 | .095 |
| Aiming for emission reduction targets provides a meaningful direction for my work in agriculture | .658 | .169 | -.023 | -.067 | -.008 | .123 | -.032 |
| I view farmers who comply with Net Zero regulations as responsible and forward-thinking professionals | .656 | -.106 | .027 | .107 | .138 | -.058 | -.140 |
| Pursuing emission reduction targets in my farming practices is a decision driven by my own values and convictions | .654 | .281 | .026 | -.089 | -.085 | -.045 | -.009 |
| Working on climate adaptation and resilience contributes to my growth as a knowledgeable and forward-thinking farmer | .652 | -.203 | -.004 | .290 | .062 | -.032 | .102 |
| The long-term benefits of investing in climate adaptation and resilience outweigh the initial costs or efforts required | .644 | -.240 | .016 | .287 | -.071 | .151 | .026 |
| Adhering to agricultural regulations aligns with my values and beliefs about responsible farming | .643 | .075 | -.024 | -.075 | .162 | .072 | -.160 |
| Deciding to focus on emission reduction targets is a choice that reflects my independent judgment and farming philosophy | .639 | .254 | -.010 | -.014 | -.057 | -.017 | -.035 |
| I am open to adapting my farming operations to comply with new or changing agricultural regulations for Net Zero | .626 | .011 | .004 | .084 | .172 | -.077 | -.021 |
| Focusing on adaptation and resilience in farming aligns with my personal values and beliefs about sustainable agriculture | .616 | -.166 | -.008 | .295 | .052 | -.022 | .089 |
| I am open to adapting my farming operations to become more resilient against climate change impacts | .607 | -.093 | -.041 | .394 | -.112 | -.003 | .089 |
| I believe that focusing on emission reduction targets will significantly enhance the environmental sustainability of my farming operations | .603 | .036 | .011 | -.039 | -.086 | .261 | .007 |
| Complying with agricultural regulations for Net Zero brings me personal satisfaction and a sense of accomplishment | .602 | .077 | .051 | -.074 | .109 | .152 | -.018 |
| Improving my farm's adaptation to climate change and its resilience gives me a sense of purpose and direction | .600 | -.088 | .032 | .303 | .021 | .066 | .018 |
| Complying with regulations gives me a sense of contributing to a larger goal of sustainability and Net Zero | .599 | .026 | .032 | -.046 | .276 | .089 | -.092 |
| Farmers who proactively adapt to climate change and work on resilience are seen as responsible and innovative | .598 | -.202 | -.011 | .287 | -.117 | .142 | .114 |
| I am open to adopting and experimenting with new technologies and support mechanisms that aid in achieving Net Zero goals | .597 | -.010 | .006 | .123 | -.023 | -.074 | .282 |
| Farmers who actively use innovative technologies and participate in support programs are seen as progressive and responsible | .596 | -.118 | -.018 | .075 | .052 | .054 | .189 |
| I have a positive attitude towards adhering to regulatory frameworks in my farming operations for Net Zero | .590 | .107 | -.007 | -.105 | .158 | .089 | -.016 |
| I find personal satisfaction and interest in exploring and adopting innovative technologies and support systems for Net Zero | .571 | -.034 | .050 | -.117 | .015 | .021 | .447 |
| I view farmers who effectively work towards emission reduction targets as role models in sustainable agriculture | .559 | .019 | -.002 | .003 | .013 | .114 | -.072 |
| I believe that adopting innovative technologies and participating in support programs will significantly enhance the efficiency and sustainability of my farming operations. | .558 | -.140 | -.028 | .095 | .039 | -.023 | .272 |
| Navigating and complying with regulatory frameworks contributes to my development as a knowledgeable and sustainable farmer | .553 | .080 | .002 | .057 | .224 | .002 | -.135 |
| Engaging with innovative technologies and support services aligns with my values and beliefs about modern and sustainable farming | .551 | .068 | .030 | -.041 | .086 | -.084 | .317 |
| The long-term benefits of complying with Net Zero regulations justify the costs and efforts involved | .548 | .048 | .023 | -.080 | .092 | .068 | .076 |
| I choose to comply with agricultural regulations as a reflection of my commitment to sustainable farming practices | .530 | .160 | .019 | .041 | .237 | .036 | -.138 |
| I believe that engaging in monitoring and reporting for Net Zero will enhance the credibility and sustainability of my farming operations | .498 | -.074 | .052 | .105 | .299 | -.033 | .064 |
| I believe that adhering to the agricultural regulatory frameworks will enhance the sustainability of my farming operations | .492 | -.007 | -.029 | .058 | .127 | .241 | -.067 |
| The long-term environmental and financial benefits of meeting emission reduction targets outweigh the initial costs | .481 | .046 | -.041 | -.047 | .014 | .088 | .107 |
| Farmers who consistently monitor and report their practices are viewed as responsible and environmentally conscious | .460 | -.195 | -.005 | .135 | .278 | .115 | .029 |
| I voluntarily comply with agricultural regulations as part of my commitment to sustainable and responsible farming | .456 | .158 | -.003 | .061 | .201 | .054 | -.068 |
| I am ready to start using new technologies and participate in support programs for Net Zero as opportunities arise | .435 | .152 | .032 | -.051 | .174 | -.042 | .264 |
| The benefits of maintaining accountability in my farming practices, such as potential recognition and trust, outweigh the effort required for monitoring and reporting. | .422 | .046 | -.016 | .002 | .248 | -.060 | .130 |
| I use information about regulations to make informed and compliant decisions in my farming operations | .327 | .256 | -.058 | .046 | .304 | -.062 | -.074 |
| Factor 2: Sustainable Farming Readiness and Confidence | | | | | | | |
| Information on how to achieve emission reduction targets is readily available and accessible to me | -.066 | .770 | -.036 | .159 | -.253 | -.017 | .119 |
| I have a clear and detailed strategy to integrate emission reduction targets into my everyday farming operations | .014 | .757 | .012 | -.122 | -.031 | .135 | .027 |
| I clearly understand the guidelines and requirements related to emission reduction targets | -.036 | .728 | -.017 | .038 | -.131 | .022 | .140 |
| I have a clear plan for how to integrate regulatory requirements into my daily farming operations for Net Zero | .019 | .661 | -.008 | -.070 | .157 | .144 | -.083 |
| I have access to the necessary resources and support to meet emission reduction targets in my farming operations. | -.254 | .633 | -.012 | .110 | -.056 | .112 | .052 |
| I am confident in my skills and abilities to implement practices that meet emission reduction targets | .200 | .632 | .015 | .110 | -.102 | -.170 | .104 |
| I have access to the necessary resources, information, and support to meet the requirements of Net Zero regulations | -.223 | .621 | .001 | .186 | .020 | .124 | .083 |
| I clearly understand the legal requirements and implications of the regulatory frameworks for Net Zero | -.030 | .608 | -.020 | .060 | .132 | .080 | -.055 |
| I can easily access information about agricultural regulations and how they contribute to achieving Net Zero | -.050 | .589 | .003 | .162 | .048 | .011 | .062 |
| I feel skilled and capable in adopting practices that align with achieving emission reduction targets. | .314 | .582 | .025 | .186 | -.109 | -.135 | -.059 |
| I have developed strategies to address potential challenges in adhering to Net Zero regulations | .034 | .577 | .005 | -.060 | .157 | .152 | -.053 |
| I have thought through potential challenges in meeting emission reduction targets and have plans to address them | .296 | .552 | .067 | -.048 | -.144 | .066 | .046 |
| I feel competent in managing my farm in a way that contributes effectively to emission reduction targets | .317 | .547 | -.005 | .107 | -.034 | -.113 | -.064 |
| Working towards emission reduction targets is becoming a routine and integral part of my farming operations. | .248 | .543 | -.017 | -.024 | -.104 | .109 | .055 |
| I am capable of critically assessing and applying information related to emission reduction targets in my farming | .270 | .516 | -.058 | .157 | -.106 | -.203 | .052 |
| I feel capable and proficient in managing my farm according to the regulatory standards set for Net Zero | .235 | .501 | -.019 | .032 | .199 | -.088 | -.021 |
| I have access to the necessary resources, training, and technical support to adopt new technologies and practices for Net Zero | -.272 | .486 | -.016 | .164 | .010 | .162 | .376 |
| I feel capable and prepared to meet the regulatory requirements for Net Zero in my farming practices | .137 | .472 | -.003 | .136 | .198 | -.045 | .008 |
| I feel skilled and knowledgeable in implementing practices that align with agricultural regulations for Net Zero | .129 | .467 | .040 | .093 | .167 | -.063 | .089 |
| I find it straightforward to understand and implement practices aimed at meeting emission reduction targets on my farm | .284 | .466 | -.043 | -.075 | -.075 | .065 | .060 |
| I have access to the necessary tools, resources, and support to effectively monitor and report my practices for Net Zero compliance | -.282 | .428 | -.058 | .297 | .235 | .075 | .085 |
| I have a clear plan for integrating innovative technologies and support services into my Net Zero strategies | -.013 | .416 | .009 | -.077 | .164 | .165 | .296 |
| Following the regulatory frameworks for Net Zero has become a routine part of my farming practices | .150 | .403 | -.013 | -.037 | .258 | .197 | -.082 |
| I can easily access information and guidelines about the monitoring and reporting requirements for Net Zero | -.151 | .394 | -.023 | .141 | .274 | .049 | .176 |
| I am capable of critically evaluating and understanding the relevance of regulatory changes to my farm | .275 | .387 | -.013 | .237 | .067 | -.207 | -.005 |
| I find it straightforward to understand and comply with agricultural regulations aimed at achieving Net Zero | .158 | .372 | -.047 | .046 | .107 | .094 | .025 |
| Factor 3: Sustainable Incentive Engagement and Acceptance | | | | | | | |
| Utilizing these financial incentives reinforces my commitment to a sustainable farming future | .064 | -.042 | .705 | -.105 | -.060 | -.123 | .090 |
| I feel capable of effectively managing financial incentives to benefit my farm's environmental practices | .000 | -.119 | .685 | .168 | -.047 | -.074 | -.059 |
| Accessing financial incentives for Net Zero contributes to my growth and development as a sustainable farmer | .002 | -.022 | .673 | .021 | -.107 | -.074 | .040 |
| I am capable of assessing the relevance and benefits of financial incentives for my farm's transition to Net Zero | .003 | -.047 | .637 | .088 | .090 | -.017 | -.080 |
| My engagement with financial incentives for Net Zero enhances my connection with the farming community and environmental initiatives | -.100 | -.061 | .635 | .079 | .020 | .100 | .027 |
| Taking advantage of financial incentives aligns with my views on responsible and sustainable farming | .106 | -.046 | .635 | -.129 | -.119 | -.093 | .067 |
| Applying for and using financial incentives for Net Zero reflects my autonomous decision-making in farm management | .050 | -.076 | .634 | .035 | .044 | -.052 | .066 |
| I have a positive view of using financial incentives to support Net Zero practices in agriculture | .089 | -.073 | .626 | -.045 | -.066 | -.085 | .021 |
| 20.14. I am ready to adapt my farming operations to qualify for financial incentives under Net Zero policies | .069 | -.072 | .619 | .018 | .084 | -.026 | -.099 |
| I independently choose to pursue financial incentives as part of my strategy for achieving Net Zero | -.034 | .054 | .615 | -.031 | .026 | -.121 | .023 |
| I actively use available information to maximize the benefits of financial incentives in my Net Zero efforts | .097 | .025 | .612 | -.026 | .020 | .039 | -.002 |
| There is a general consensus among those important to me that taking advantage of Net Zero financial incentives is beneficial | .036 | -.100 | .591 | -.057 | .029 | .084 | -.016 |
| I feel competent and knowledgeable in navigating the financial aspects of Net Zero incentives | -.210 | .113 | .587 | .174 | .121 | -.057 | -.032 |
| I am willing to modify my farming practices to meet the criteria for Net Zero financial incentives. | .160 | -.135 | .583 | -.053 | -.201 | -.117 | .177 |
| Utilizing financial incentives for Net Zero practices is becoming a standard part of my financial planning for the farm | .052 | .067 | .578 | -.065 | .061 | -.031 | -.081 |
| Engaging with financial incentives for Net Zero has improved my relationships with other farmers and stakeholders | -.015 | .042 | .558 | -.069 | -.065 | .186 | .046 |
| Information about financial incentives for Net Zero practices is readily accessible to me | -.107 | .096 | .558 | .063 | .055 | .026 | -.043 |
| The financial benefits provided by Net Zero incentives make the transition to sustainable practices more appealing | .031 | -.040 | .555 | -.040 | -.021 | .025 | -.028 |
| I have strategies in place to navigate the complexities of applying for and utilizing financial incentives for Net Zero | .010 | .100 | .527 | -.025 | .223 | .043 | -.106 |
| I feel encouraged by my peers and the agricultural community to take advantage of Net Zero financial incentives | -.113 | -.007 | .519 | .035 | -.032 | .228 | -.012 |
| I find personal satisfaction and motivation in leveraging financial incentives to achieve Net Zero goals | .141 | -.042 | .518 | .001 | -.097 | -.001 | -.027 |
| I feel confident in my ability to navigate and utilize the financial incentives available for Net Zero | -.047 | .072 | .516 | -.080 | .116 | -.017 | .037 |
| I clearly understand the criteria and processes involved in obtaining financial incentives for Net Zero practices | -.126 | .077 | .512 | .096 | .108 | .098 | -.057 |
| I find it easy to understand and take advantage of financial incentives available for Net Zero practices | -.129 | .191 | .494 | .021 | -.098 | .134 | -.024 |
| I have the necessary information and support to access financial incentives for Net Zero practices | -.257 | .245 | .482 | .130 | .027 | .061 | -.042 |
| I have a clear plan for how to incorporate financial incentives into my Net Zero strategies | -.055 | .165 | .478 | .037 | .122 | .071 | -.058 |
| I believe that utilizing financial incentives for Net Zero will significantly improve the sustainability and profitability of my farming operations | .008 | -.013 | .464 | -.045 | -.174 | .047 | .023 |
| Farmers who successfully utilize financial incentives are seen as savvy and environmentally conscious | .065 | -.145 | .459 | -.114 | .017 | -.083 | .073 |
| Factor 4: Climate Adaptation Competence and Confidence in Agriculture | | | | | | | |
| I feel skilled and effective in implementing strategies that enhance my farm's adaptation to climate change and resilience | .070 | .214 | -.016 | .646 | .045 | -.030 | -.032 |
| I feel confident in my ability to implement adaptation and resilience practices in response to climate change | .246 | .115 | -.003 | .639 | -.038 | -.130 | -.026 |
| I am capable of evaluating and applying information related to climate adaptation and resilience in my farming | .234 | .029 | -.048 | .611 | .167 | -.135 | -.040 |
| I clearly understand the concepts and practices related to agricultural adaptation to climate change and resilience building | .112 | .131 | .004 | .596 | .037 | .032 | -.003 |
| I feel capable of effectively managing my farm with practices that enhance adaptation to climate change and resilience | .297 | .110 | .033 | .588 | .069 | -.127 | -.074 |
| I can easily access information on climate adaptation and resilience strategies for agriculture | .058 | .217 | .025 | .543 | -.012 | .036 | .043 |
| I actively use available information to make my farming operations more adapted and resilient to climate change | .136 | .134 | .011 | .500 | .084 | .069 | .068 |
| I find the process of adapting my farming practices for greater resilience to climate change to be manageable and straightforward | .191 | .141 | -.034 | .425 | .024 | .161 | -.065 |
| I have strategies in place to address potential challenges in adapting to climate change and enhancing resilience | .074 | .308 | .065 | .390 | .064 | .111 | .027 |
| Factor 5: Net Zero Accountability and Reporting Commitment | | | | | | | |
| I am prepared to start or enhance my monitoring and reporting processes to meet Net Zero requirements | .291 | -.024 | .001 | .089 | .579 | -.023 | -.038 |
| I autonomously engage in monitoring and reporting practices as a self-driven decision towards responsible farming | .206 | .045 | -.015 | -.021 | .570 | -.007 | .049 |
| I feel capable of managing my farm in a way that meets the accountability standards set for Net Zero | .268 | .091 | -.002 | .163 | .548 | -.207 | -.013 |
| I effectively use the information and guidelines provided to ensure my farming practices meet Net Zero accountability standards | .125 | .104 | .008 | .039 | .544 | .097 | .013 |
| I am confident in my ability to adhere to the required monitoring and reporting standards for Net Zero | .051 | .186 | -.008 | .100 | .542 | -.057 | .062 |
| I have a positive attitude towards the accountability measures (monitoring, reporting, verifying) required for Net Zero | .347 | -.069 | .017 | -.091 | .523 | .010 | .134 |
| Regular monitoring and reporting of my farming practices for Net Zero have become a routine part of my farm management | .017 | .220 | .021 | -.067 | .512 | .113 | .086 |
| I feel competent in implementing and managing the monitoring and reporting processes required for Net Zero | .082 | .240 | -.022 | .166 | .474 | -.083 | .093 |
| I am capable of evaluating and integrating information related to accountability measures for Net Zero in my farm operations | .056 | .110 | -.014 | .266 | .449 | -.137 | .073 |
| I find a sense of personal satisfaction in knowing that my farming practices are monitored and reported for Net Zero compliance. | .312 | .005 | -.011 | -.074 | .431 | .088 | .122 |
| My adherence to accountability standards has improved trust and relationships with stakeholders and the community | .154 | .078 | -.006 | -.055 | .429 | .235 | .093 |
| I clearly understand the requirements and processes for monitoring, reporting, and verifying my practices for Net Zero | -.163 | .286 | -.020 | .153 | .417 | .081 | .116 |
| I find the process of monitoring, reporting, and verifying my farming practices for Net Zero straightforward and manageable | .063 | .233 | -.019 | .057 | .396 | -.021 | .147 |
| I feel a sense of responsibility from my peers and the agricultural community to accurately report and verify my farming practices for Net Zero | .215 | .052 | -.047 | .066 | .330 | .281 | -.066 |
| Factor 6: Community Influence and Commitment in Sustainable Farming | | | | | | | |
| I feel influenced by my peers and the agricultural community to comply with Net Zero regulatory frameworks | .075 | .249 | -.031 | -.137 | .084 | .529 | -.028 |
| I feel influenced by my peers and the agricultural community to focus on climate adaptation and resilience in my farming practices | .167 | .040 | -.007 | .227 | .056 | .496 | -.090 |
| I feel motivated by my peers and agricultural experts to engage with innovative technologies and support programs for Net Zero | .169 | .057 | -.020 | .014 | .056 | .491 | .239 |
| Working towards emission reduction targets has strengthened my relationships with other environmentally conscious farmers | .271 | .278 | .014 | -.119 | -.033 | .488 | -.039 |
| My focus on climate adaptation and resilience has improved my relationships with other farmers, experts, and the community | .214 | .032 | .016 | .259 | -.039 | .457 | .073 |
| My engagement with innovative technologies and support programs has improved my relationships with other farmers and industry experts | .090 | .191 | .072 | .025 | .025 | .435 | .225 |
| I feel a strong sense of responsibility from my community to adhere to emission reduction targets | .254 | .313 | -.034 | -.030 | -.132 | .415 | -.019 |
| I feel that it is expected of me by my community and other stakeholders to follow agricultural regulations for Net Zero | .166 | .194 | -.069 | .015 | .059 | .405 | .000 |
| My commitment to emission reduction targets has deepened my sense of connection with the farming community and the environment | .298 | .302 | .043 | -.076 | -.007 | .373 | .005 |
| My adherence to regulatory frameworks has enhanced my sense of belonging and collaboration within the agricultural community | .230 | .256 | .000 | .011 | .168 | .344 | -.043 |
| Factor 7: Innovation and Technological Competence in Net Zero Farming | | | | | | | |
| There is a general consensus among important stakeholders that engaging with innovation and support programs is beneficial for achieving Net Zero | .248 | .003 | -.037 | .057 | .073 | .091 | .461 |
| I feel skilled and innovative in using new technologies and accessing support services to meet Net Zero goals | .175 | .279 | -.002 | .103 | .097 | -.139 | .432 |
| Incorporating innovative technologies and support mechanisms into my farming operations for Net Zero is becoming a routine practice | .137 | .279 | .046 | -.068 | .106 | .157 | .404 |
| I feel capable and equipped to utilize innovative technologies and support services for Net Zero | .080 | .308 | .019 | .171 | .091 | -.176 | .390 |
| I clearly understand the benefits and applications of new technologies and support mechanisms for achieving Net Zero | .157 | .206 | -.060 | .045 | .167 | -.002 | .371 |
| I actively apply information about new technologies and support services to enhance my farm's Net Zero efforts | .211 | .296 | .024 | -.016 | .122 | .001 | .359 |
| I feel competent and effective in managing my farm using innovative technologies and support services for Net Zero | .171 | .250 | .003 | .195 | .140 | -.160 | .352 |
| **Mixed loadings** | | | | | | | |
| I feel motivated by my peers and community to adopt practices that align with emission reduction targets. | .340 | .263 | .038 | -.056 | -.104 | .323 | .014 |
| I am prepared to immediately start implementing practices that contribute to emission reduction targets | .402 | .448 | .034 | .065 | -.100 | .074 | -.116 |
| I actively use the information available about emission reduction targets to make informed decisions on my farm | .333 | .545 | .040 | -.067 | -.136 | .049 | .049 |
| I am ready to implement changes required by regulatory frameworks for Net Zero as needed | .346 | .350 | -.010 | -.060 | .244 | -.035 | -.058 |
| Complying with regulations has strengthened my relationships within the farming community and with regulatory bodies | .177 | .372 | .012 | -.096 | .008 | .419 | .076 |
| I am willing to adapt my farming operations to ensure compliance with monitoring and reporting requirements for Net Zero | .466 | -.057 | .017 | -.099 | .560 | -.043 | .052 |
| I have a clear plan and system in place for the regular monitoring and reporting of my farming practices for Net Zero | -.064 | .369 | -.026 | -.037 | .480 | .087 | .057 |
| I have strategies to manage and overcome challenges related to monitoring and reporting for Net Zero | -.039 | .325 | .019 | .058 | .553 | .039 | -.040 |
| Engaging in accountability measures for Net Zero aligns with my values and beliefs about responsible farming | .620 | -.115 | .005 | -.062 | .417 | -.062 | .063 |
| Participating in monitoring and reporting practices contributes to my growth as a knowledgeable and responsible farmer | .454 | -.164 | -.011 | -.025 | .574 | .029 | -.038 |
| My commitment to accountability in farming practices reflects my dedication to a sustainable agricultural future | .453 | -.183 | .012 | -.023 | .582 | -.028 | .026 |
| I choose to engage in accountability practices as part of my commitment to sustainable and transparent farming | .379 | -.090 | .029 | -.031 | .545 | .085 | .005 |
| My commitment to accountability in farming enhances my connection with the broader agricultural community and environment-focused initiatives | .131 | .067 | -.023 | .053 | .409 | .332 | .052 |
| I find it easy to understand and integrate new technologies and support programs aimed at achieving Net Zero into my farming practice | .090 | .327 | -.046 | .035 | -.019 | .045 | .449 |
| The benefits of investing in new technologies and participating in support programs for Net Zero outweigh the costs | .414 | .025 | .002 | -.125 | -.026 | .139 | .449 |
| I have a positive view of using innovative technologies and participating in support programs for Net Zero in agriculture | .569 | -.086 | .032 | -.062 | .050 | .027 | .421 |
| I have strategies to manage challenges associated with adopting new technologies and support services for Net Zero | .033 | .447 | .037 | -.059 | .142 | .020 | .366 |
| Information about new technologies and support programs for Net Zero is readily accessible to me. | -.128 | .466 | .012 | .063 | .079 | .058 | .386 |
| Adopting new technologies and participating in support programs contributes to my personal and professional development as a farmer | .564 | -.016 | -.042 | -.058 | .108 | -.089 | .369 |
| Using innovative solutions and support services for Net Zero gives a meaningful direction to my agricultural practices | .526 | -.055 | -.028 | -.086 | .115 | .022 | .429 |
| I choose to engage with new technologies and support services as part of my proactive approach to sustainable farming | .328 | .114 | .050 | .039 | .111 | .061 | .354 |
| I voluntarily engage with new technologies and support programs as a self-driven decision to advance my farm towards Net Zero | .351 | .158 | .025 | -.046 | .099 | .000 | .432 |
| My involvement with innovative technologies and support initiatives enhances my connection with the progressive agricultural community and environmental movements | .178 | .068 | -.031 | -.006 | .112 | .330 | .386 |
| I believe that adapting to climate change and enhancing resilience will significantly improve the sustainability of my farming operations | .576 | -.187 | -.017 | .348 | -.065 | .098 | .052 |
| Integrating climate adaptation and resilience measures into my farming operations is becoming a routine part of my management strategy | .350 | .094 | .025 | .411 | .006 | .180 | -.059 |
| I feel a sense of responsibility from the farming community to enhance my farm’s adaptation and resilience to climate impacts | .401 | -.027 | .022 | .389 | -.206 | .398 | .013 |
| I have a clear and detailed plan to integrate climate adaptation and resilience practices into my farming operations | -.010 | .352 | -.019 | .380 | .038 | .200 | .002 |
| I am ready to start or enhance my practices to adapt to climate change and improve resilience | .347 | .035 | .047 | .488 | .068 | .069 | -.082 |
| I independently choose to implement adaptation and resilience practices as part of my commitment to sustainable farming | .356 | -.007 | -.021 | .471 | .156 | .035 | -.124 |
| I autonomously engage in climate adaptation and resilience practices as part of my decision-making for sustainable farming | .390 | .044 | .011 | .361 | .014 | .083 | .043 |
| My efforts in climate adaptation and resilience strengthen my connection with the agricultural community and environmental initiatives | .195 | .101 | .016 | .335 | -.062 | .412 | .051 |
| I have access to the necessary resources, information, and support to effectively adapt my farm to climate change and enhance resilience | -.162 | .351 | .000 | .502 | -.076 | .209 | .039 |
| **No Loadings** | | | | | | | |
| I am capable of evaluating the relevance and effectiveness of new technologies and support programs for my farm's transition to Net Zero | .179 | .210 | -.042 | .234 | .146 | -.182 | .299 |
| I feel that complying with monitoring and reporting requirements is expected and respected in the farming community | .182 | .033 | -.021 | -.030 | .313 | .290 | .158 |
