# Supplementary material for: Avoiding siloed approaches: Integrating psychological insights into sustainable farming
Source: PLoS One. 2024 Oct 14;19(10):e0301881. doi: 10.1371/journal.pone.0301881 (PMC11472944; doi:10.1371/journal.pone.0301881)
Supplement: Attachment — Submitted filename: Comments to author Plos one.docx [file pone.0301881.s002.docx]

Avoiding Silo Thinking: Integrating Psychological Insights into Farming Net Zero Policy Commitments

**Abstract:** The study presented offers a comprehensive examination of the psychological drivers influencing farmers' adoption of Net Zero policies, incorporating a wide range of theoretical frameworks. This multi-dimensional approach, encompassing theories such as the Unified Theory of Acceptance and Use of Technology and Self-Determination Theory, enriches our understanding of farmers' attitudes and behaviors towards sustainability initiatives. However, while the integration of diverse psychological perspectives is commendable, it may be beneficial to provide a clearer rationale for the selection of specific theories and how they interact within the context of sustainable agriculture. Additionally, the findings from Study 1, elucidating seven latent factors influencing farmers' engagement with Net Zero policies, provide valuable insights into the nuanced motivations and challenges faced by agricultural practitioners. Nevertheless, further elaboration on the operationalization of these factors and their practical implications for policy development and intervention strategies would enhance the study's applicability and relevance to stakeholders in the agricultural sector. Moreover, Study 2's validation of the proposed psychological model through Confirmatory Factor Analysis and concurrent validity assessment represents a significant contribution to the field. However, considering the geographical variation in agricultural practices, particularly between the UK and the US, it would be informative to explore potential cross-cultural differences in the psychological determinants of Net Zero farming. Overall, this research offers a robust framework for understanding the complex interplay of psychological factors shaping farmers' adoption of sustainable practices, laying the groundwork for future investigations and practical interventions aimed at promoting environmental stewardship in agriculture.

**Introduction:** The passage effectively integrates a wide array of psychological frameworks to elucidate the complexities of farmers' adoption of sustainable practices. However, to enhance clarity and coherence, it could benefit from a more structured presentation of these frameworks, perhaps organized into distinct sections or subsections based on thematic similarities or theoretical foundations. This structural refinement would assist readers in navigating the diverse theoretical perspectives and their interconnections more seamlessly.

Furthermore, while the passage highlights the importance of simplifying and unifying psychological frameworks for practical application, it could provide more concrete examples or case studies illustrating how this integration process could be operationalized in real-world contexts. By demonstrating the application of the proposed unified model in specific agricultural settings or initiatives, readers would gain a clearer understanding of its potential implications and utility for stakeholders in the field.

Additionally, the passage could delve deeper into the potential challenges or limitations associated with integrating multiple psychological frameworks, such as conflicting theoretical assumptions or methodological differences. Acknowledging and addressing these challenges would add depth to the discussion and provide a more nuanced understanding of the complexities involved in developing a comprehensive psychological model for sustainable agriculture.

Finally, while the passage outlines the methodological approach for developing and validating the proposed psychological model, it could provide more detail on the specific steps involved in data collection, analysis, and interpretation. Providing transparency regarding the research methodology would enhance the credibility and replicability of the study, enabling readers to assess the rigor of the findings and their applicability to other contexts.

The discussion effectively outlines the seven-factor model and the development of the ANZAR Scale, providing valuable insights into the psychological dimensions of farmers' engagement with Net Zero practices. However, to enhance clarity and coherence, it could benefit from a more structured presentation, perhaps organizing the discussion around each factor or scale development process individually. This would help readers navigate the complex interplay of psychological theories and empirical findings more seamlessly, facilitating a deeper understanding of the research outcomes.

Additionally, while the discussion highlights the practical implications of the findings for policymakers and practitioners, it could provide more specific examples or case studies illustrating how the ANZAR Scale could be utilized in real-world contexts. Concrete examples demonstrating how the scale could inform the design of targeted interventions or support mechanisms tailored to farmers' psychological profiles would enhance the applicability of the research findings and offer actionable insights for stakeholders in the agricultural sector.

Furthermore, the discussion acknowledges the limitations of the study, such as its cross-sectional nature and the need for future research to explore longitudinal designs and regional policy environments. While these limitations are appropriately addressed, providing suggestions for how future studies could mitigate or overcome these limitations would add depth to the discussion and guide researchers in designing more robust investigations in the future.

Finally, the discussion could emphasize the importance of interdisciplinary collaboration in advancing research on sustainable agriculture. Highlighting the potential benefits of integrating insights from disciplines such as psychology, agronomy, economics, and environmental science could underscore the need for a holistic approach to addressing the complex challenges of achieving Net Zero emissions in agriculture.

In summary, while the discussion provides a thorough examination of the research findings and their implications, further improvements could be made to enhance the clarity, applicability, and interdisciplinary perspective of the discussion. By incorporating these suggestions, the discussion could offer a more comprehensive and impactful contribution to the field of sustainable agriculture.
