# Supplementary material for: Avoiding siloed approaches: Integrating psychological insights into sustainable farming
Source: PLoS One. 2024 Oct 14;19(10):e0301881. doi: 10.1371/journal.pone.0301881 (PMC11472944; doi:10.1371/journal.pone.0301881)
Supplement: Attachment — Submitted filename: Response to Reviewers.docx [file pone.0301881.s003.docx]

**RESPONSE TO REVIEWERS**

Thank you for considering the recent submission and for your encouraging response regarding the revision and resubmission of the manuscript. I extend my gratitude to the reviewers for their insightful, helpful, patient, and constructive feedback. In response to the reviewers' comments, I have thoroughly reviewed the paper and made numerous significant changes and additions. I have also corrected some typing errors and I apologise for the presence of those. In also revising the paper, and making corrections, I have provided the exact dates for data collection as submission guidelines and amended the name of the scale (to the **Integrated Motivation Model for Sustainable Farming scale),** to make it more concordant with the revisions. To provide clarity the revised text corresponding to each response is included alongside the response with the location of the change in the revised manuscript also presented.

**Additional Editor Comments**

**1. Please improve the abstract, Introduction and Disucssion section. Abstract is very technical. It should reflect the data sources, methods, and the main findings of the study as well the policy implications. Furthermore, clarify what is silo thinking and improve upon it in the Introduction section while accentuating the research gap and relevant literature. State the obejctives clearly. For improvement in Discussion section, see reviewers' comments.**

>>>>> We have rewritten the abstract complete and it reads on Page 2, Line 2 to Page 3. Line 2 of the revised manuscript as follows:

“This study enhances our understanding of the psychological factors influencing farmers' adoption of sustainable farming practices, specifically those related to achieving NetZero emissions. It achieves this by integrating various psychological theories with practical farming methods within the context of Behavioral-Adoption and Purpose-Driven contexts. The research consisted of two studies. Study 1 employed Exploratory Factor Analysis (EFA) to analyze responses from 438 UK farmers regarding their attitudes toward a series of Net Zero policy commitments, drawing on psychological theories including the Unified Theory of Acceptance and Use of Technology, the Theory of Planned Behavior, the Prototype Willingness Model, Implementation Intentions, Self-Determination Theory, Eudaimonia, and the Integrated Model of Health Literacy. The findings revealed a new Integrated Motivation Model for Sustainable Farming that comprises seven factors: Agricultural Commitment and Stewardship, Sustainable Farming Readiness and Confidence, Sustainable Incentive Engagement and Acceptance, Climate Adaptation Competence and Confidence, Net Zero Accountability and Reporting Commitment, Community Influence and Commitment in Sustainable Farming, and Innovation and Technological Competence. Study 2 validated these factors through the development of a 21-item Integrated Motivation Model for Sustainable Farming scale and use of Confirmatory Factor Analysis (CFA) to confirm the 7-factor structure using a subsample of 418 UK farmers from Study 1 and an additional 230 US farmers. Furthermore, Study 2 tested the concurrent validity of the new scale by demonstrating significant associations with reported sustainable farming behaviors. These findings underscore the complex interplay of motivational, cognitive, and social processes influencing sustainable farming practices. The integrated psychological model developed through this research provides parsimonious and valuable insights for policymakers to foster sustainable practices in farming effectively. The confirmation of this model across farming populations enhances its generalizability and potential to guide targeted interventions aimed at achieving behavioral change in pursuit of Net Zero targets in agriculture.”

**2. Furthermore, clarify what is silo thinking and improve upon it in the Introduction section while accentuating the research gap and relevant literature.**

>>>>> In response to the reviewer's suggestion, we have expanded the discussion of 'silo thinking' – though we’ve re-termed it has “silo approaches” in the Introduction to clarify its meaning and implications within the context of sustainable agriculture. This revision appears on Page 6, Lines 18 to 21 of the revised manuscript.

“By identifying common themes across these frameworks, we can simplify complex theories into practical strategies that prevent siloed approaches (an approach where different sectors or disciplines operate in isolation) and enhance farmers' and land managers' adoption of sustainable practices.”

We have also enhanced the section by explicitly linking the concept to the identified research gap and discussing relevant literature, a revision also largely shaped by the first comment from Reviewer 2. This amendment not only addresses the reviewer's concerns but also strengthens the manuscript by providing a clearer exposition of the theoretical underpinnings of our research. It demonstrates the necessity and impact of a multidisciplinary approach in addressing complex agricultural challenges, incorporating Behavioral-Adoption and Purpose-Driven frameworks. We have added this aspect to the paragraph. This revision appears from Page 6, Line 21 to Page 7, Line 14 of the revised manuscript.

“This integration helps bridge the gap between intricate psychological theories and farming realities, promoting a more cohesive understanding. For example, in regions where farming demands significant water resources, leading to environmental stress, especially during drought conditions, promoting water-efficient technologies poses a challenge (21). Farmers may be hesitant due to high upfront costs, uncertainty about effectiveness, and disruption of established practices. A better understanding can be achieved by integrating factors from Behavioral-Adoption and Purpose-Driven frameworks. We could identify factors that emphasize practical readiness and external factors such as financial incentives (Behavioral-Adoption), which align with farmers' values towards sustainability and stewardship of natural resources (Purpose-Driven). This integration provides a more meaningful and strategic understanding of these factors. Similarly, in areas where farming is crucial to the economy and vulnerable to climate change (22), a unified model that integrates Behavioral-Adoption strategies—demonstrating the effectiveness and efficiency of new sustainable practices—with the Purpose-Driven aspects that engage farmers' values and cultural practices related to land and community welfare, can be advantageous. This dual approach ensures farmers are not only prepared but also motivated to adopt new practices that benefit both their livelihood and their community. Thus, this unified approach addresses both the logistical and motivational aspects of adopting or changing behaviors to target sustainable farming.”

**3. State the objectives clearly.**

>>>>> In response to the reviewer’s comment, we have clarified the objectives of our study by explicitly listing them and detailing the methodological approaches used to achieve each goal. The revision includes a structured breakdown of the study's aims, enhancing the clarity and readability of our research intentions. This change addresses the need for a precise and comprehensible presentation of our study’s goals, ensuring that the objectives are unmistakably clear and logically presented. This amendment will aid in better understanding the scope and direction of our research within the broader context of psychological studies in sustainable agriculture. This revision appears on Page 8, Line 11 to Page 9, Line 5 of the revised manuscript.

“The primary aim of this study is to develop a comprehensive psychological model that merges the Behavioral-Adoption and Purpose-Driven frameworks around sustainable farming practices to achieve NetZero among farmers. This integrated model will enhance our understanding of the psychological factors that influence farmers’ behaviors and motivations towards sustainable farming practices to achieve NetZero goals. Specifically, there are three objectives:

1. Develop an integrated psychological framework that integrates diverse perspectives to explain behavior change in the context of sustainable farming. This framework will utilize exploratory factor analysis (EFA) as an abductive reasoning method (26) to identify underlying psychological factors from a broad array of existing psychological models and constructs that reflect Behavioral-Adoption and Purpose-Driven frameworks.
2. Demonstrate structural validity for the proposed integrated psychological model through confirmatory factor analysis (CFA) (27), , ensuring that our model reliably captures the essential motivational, cognitive, and social processes that influence farmers’ decisions.
3. Assess the model’s practical implications by linking the psychological factors to actual behavioral indicators of NetZero farming practices, thereby demonstrating the model’s concurrent validity.”

**4. For improvement in Discussion section, see reviewers' comments.**

>>>>> "In our revised manuscript, we have taken on board the reviewers' comments, which are detailed below. We have elaborated on the operationalization of factors affecting net zero emissions in agriculture, providing detailed practical implications for policy development and intervention strategies. We structured the discussion around individual factors and the scale development process to enhance understanding of the interplay between psychological theories and empirical findings. Our study addresses its limitations and offers suggestions for future research to build more robust methodologies. Additionally, we suggest that this provides confidence for future work looking towards integrated insights from psychology, agronomy, economics, and environmental science, advocating for a holistic approach to tackle the complex challenges of achieving net zero emissions in the agricultural sector.

**Reviewer #1:**

1. **However, the second paragraph is a bit too long and could be shortened by combining some of the sentences.**

>>>>> Thank you for the suggestion. Due to other comments, we have rewritten the introduction, so the structure and content are different – in which we make a case for grouping psychological theories into behavioural adoption and purpose-drive frameworks. Nonetheless, we have been mindful about the length of paragraphs.

2. **Additionally, the third paragraph could be improved by adding a few more specific examples.**

>>>>> In the revised manuscript, we have structured the work into Behavioural-adoption and Purpose-driven theories. This paragraph – which is now the fourth - is now part of the purpose-driven theories. However, I have expanded this paragraph by adding more specific examples of how theories like the Theory of Implementation Intentions can be practically applied, particularly in agricultural contexts. Examples include the successful implementation of detailed action plans for sustainable practices like pesticide reduction and water conservation in diverse geographical areas. This addition aims to demonstrate the real-world applicability and effectiveness of the integrated theoretical models discussed, thereby addressing your valuable suggestion to enhance the specificity of examples provided in the manuscript. This revised paragraph appears on Page 5, Line 11 to Page 6, Line 13 of the revised manuscript as follows:

“The second set to consider are Purpose-Driven frameworks that focus on beliefs, meaning, fulfillment, desired prototypes, environmental literacy processes, and contributions to personal and community well-being. Within the existing sustainable farming literature, Self-Determination Theory (SDT;(7)) has been used to explore practices in sustainable farming as reflections of purpose-driven intentions in farming, in terms of motivational intrinsic versus extrinsic needs driven by autonomy, competence, and relatedness. Rsearch shows that SDT principles are instrumental in how a self-directed approach contributes to sustainable farming, emphasizing how intrinsic choices over external rewards and fostering conditions that support farmer autonomy leads to sustainable farming (8,9). Moreover, integrating models such as the Prototype Willingness Model (10) and the theory of Implementation Intentions (11,12) could offer an advanced understanding of concepts of prototypical influences around the identity of being a sustainable farmer (e.g., the ‘eco-friendly farmer’) and intentional, planned behaviors (e.g. specific plans) in sustainable farming are Purpose-Driven factors in farming. For example, in terms of implementation intentions, developing detailed action plans for pesticide reduction or water conservation can underline the practical applicability of these theories. (13,14). There are also other psychological framework that we consider Purpose-Driven. The inclusion of models of environmental literacy (15) that enriches our understanding by framing the competencies necessary for sustainable farming. This approach not only emphasises individuals' abilities to access and understand information relating to sustainable farming, but also emphasis the ability to make purposeful and well-informed decisions that positively impact on sustainable farming. Lastly, though there has been an exploration of the relationship between everyday affect (e.g. enjoyment) and farming practices (16,17) there is a compelling need for a broader examination of well-being concepts, particularly eudaimonia. Eudaimonia focuses on fulfilling one's potential and finding deep meaning and happiness in alignment with one’s true self (18,19). Exploring how eudaimonia correlates with sustainable farming practices (20) offers vital insights into possibly why fulfilment and meaning may be purposefully connected with the adoption of sustainable practices in farming.”

3. **The conclusion is well-written and summarizes the main points of the paper well. However, I would suggest adding a sentence or two about the implications of the findings.**

>>>>> In response to your suggestion, we have expanded the conclusion to include a discussion on the implications of our findings. This addition emphasizes the potential for using our results to inform targeted interventions that address both the psychological and practical aspects of sustainable farming. This revision appears on Page 36, Lines 8 to 12 of the revised manuscript.

“The findings underscore the potential for targeted interventions that leverage psychological insights to foster sustainable farming practices. By understanding the motivational and cognitive processes that influence farmers' behavior, policymakers and practitioners can design more effective strategies that not only promote environmental sustainability but also support the well-being of farmers.”

**Reviewer #2:** 

1. **The study presented offers a comprehensive examination of the psychological drivers influencing farmers' adoption of Net Zero policies, incorporating a wide range of theoretical frameworks. This multi-dimensional approach, encompassing theories such as the Unified Theory of Acceptance and Use of Technology and Self-Determination Theory, enriches our understanding of farmers' attitudes and behaviours towards sustainability initiatives. However, while the integration of diverse psychological perspectives is commendable, it may be beneficial to provide a clearer rationale for the selection of specific theories and how they interact within the context of sustainable agriculture.**

>>>>> This consideration (together with point 4 made by this reviewer below) led to major changes in the structure and positioning of the paper, during which we realized that the psychological theories needed to be organized within a wider context. Therefore, we have separated the frameworks into broadly behavioral adoption—based on existing literature—and purpose-driven, which introduces more value-based considerations. By defining these, we believe the literature is more organized, and the paper makes a more significant contribution to the field. We also feel that this helps cement many aspects of the paper in terms of providing a wider context for the interpretation of the model's findings and describing the resulting model. This revision appears on Page 4, Line 10 to Page 6, Line 13 of the revised manuscript.

“In this consideration, we present two main psychological contexts that influence farmers' decision-making towards sustainable farming: Behavioral-Adoption and Purpose-Driven frameworks. The Behavioral-Adoption frameworks, often cited in sustainable farming literature, focus on external factors like social influence and practical utility, which are crucial for the adoption of new technologies and practices by farmers. For instance, these frameworks explore how peer attitudes and the availability of supportive conditions can encourage sustainable behaviors. On the other hand, the Purpose-Driven frameworks delve into the internal motivations of farmers, such as personal fulfillment and alignment with core values, which drive their engagement in sustainable practices. These frameworks emphasize understanding why farmers choose to engage in sustainability from an intrinsic perspective. By contrasting and integrating these frameworks, our study offers a comprehensive view of the dynamic interplay between external influences and internal motivations that shape sustainable agricultural practices..

In terms of how these two sets of frameworks relate to specific psychological model and sustainable farming, the first set to consider are Behavioral-Adoption frameworks. Am exampler model is the Unified Theory of Acceptance and Use of Technology (1), which explains technology acceptance by highlighting factors like performance expectancy (the technology’s perceived usefulness), social influence (the impact of peer attitudes), and facilitating conditions (the presence of a supportive environment). It is these elements of the Unified Theory of Acceptance and Use of Technology model that significantly influences farmers’ attitudes toward adopting sustainable farming practices (2). Complementing the Unified Theory of Acceptance and Use of Technology, the Theory of Planned Behavior (3) provides a framework around adopting sustainable technologies in farming via intentions, shaped by attitudes, norms, and perceived control, predict behaviors. Research shows that attitudes, social norms, and perceived control positively influence farmers' intentions to adopt practices like organic farming and improved natural grassland management (4–6).

The second set to consider are Purpose-Driven frameworks that focus on beliefs, meaning, fulfillment, desired prototypes, environmental literacy processes, and contributions to personal and community well-being. Within the existing sustainable farming literature, Self-Determination Theory (SDT;(7)) has been used to explore practices in sustainable farming as reflections of purpose-driven intentions in farming, in terms of motivational intrinsic versus extrinsic needs driven by autonomy, competence, and relatedness. Rsearch shows that SDT principles are instrumental in how a self-directed approach contributes to sustainable farming, emphasizing how intrinsic choices over external rewards and fostering conditions that support farmer autonomy leads to sustainable farming (8,9). Moreover, integrating models such as the Prototype Willingness Model (10) and the theory of Implementation Intentions (11,12) could offer an advanced understanding of concepts of prototypical influences around the identity of being a sustainable farmer (e.g., the ‘eco-friendly farmer’) and intentional, planned behaviors (e.g. specific plans) in sustainable farming are Purpose-Driven factors in farming. For example, in terms of implementation intentions, developing detailed action plans for pesticide reduction or water conservation can underline the practical applicability of these theories. (13,14). There are also other psychological framework that we consider Purpose-Driven. The inclusion of models of environmental literacy (15) that enriches our understanding by framing the competencies necessary for sustainable farming. This approach not only emphasises individuals' abilities to access and understand information relating to sustainable farming, but also emphasis the ability to make purposeful and well-informed decisions that positively impact on sustainable farming. Lastly, though there has been an exploration of the relationship between everyday affect (e.g. enjoyment) and farming practices (16,17) there is a compelling need for a broader examination of well-being concepts, particularly eudaimonia. Eudaimonia focuses on fulfilling one's potential and finding deep meaning and happiness in alignment with one’s true self (18,19). Exploring how eudaimonia correlates with sustainable farming practices (20) offers vital insights into possibly why fulfilment and meaning may be purposefully connected with the adoption of sustainable practices in farming.”

2. **Additionally, the findings from Study 1, elucidating seven latent factors influencing farmers' engagement with Net Zero policies, provide valuable insights into the nuanced motivations and challenges faced by agricultural practitioners. Nevertheless,** **further elaboration on the operationalization of these factors and their practical implications for policy development and intervention strategies would enhance the study's applicability and relevance to stakeholders in the agricultural sector.**

>>>>> We have added two paragraphs to the discussion that do this – First in terms of the model. This revision appears on Page 30, Line 24 to Page 31, Line 24 of the revised manuscript.

“We introduce the Integrated Motivation Model for Sustainable Farming, which defines seven key factors by merging two distinct frameworks presented in this paper: Behavioral-Adoption and Purpose-Driven. The Behavioral-Adoption framework is focused on enhancing practical readiness, skill development, and strategic implementation necessary for adopting sustainable practices. It emphasizes external motivations such as incentives, compliance with standards, and new technology utilization. The five factors identified in the study—Sustainable Farming Readiness and Confidence, Sustainable Incentive Engagement and Acceptance, Climate Adaptation Competence and Confidence in Agriculture, Net Zero Accountability and Reporting Commitment, and Innovation and Technological Competence in Net Zero Farming—represent this framework. They underscore the importance of equipping farmers with the necessary tools and information, creating environments conducive to sustainable practices, and ensuring farmers feel competent and supported. Conversely, the Purpose-Driven framework delves into intrinsic and value-based motivations driving sustainable farming. It aligns farmers’ actions with their personal values and community norms, fostering deeper engagement and commitment to sustainability. Factors such as Sustainable Agricultural Commitment and Stewardship, and Community Influence and Commitment in Sustainable Farming reflect this framework, focusing on internal satisfaction and personal fulfillment driven by a responsibility towards the community and environment. The interplay of these frameworks within our model offers a dual perspective that captures the motivational, cognitive, and social processes influencing sustainable farming. This integration helps us understand not only the practical steps necessary for behavioral adoption but also the deeper sense of purpose sustaining these changes over time. By illuminating the complex interplay between Behavioral-Adoption and Purpose-Driven factors, our model provides a holistic view of what drives farmers towards achieving Net Zero goals. It becomes a crucial tool for researchers, policymakers, and practitioners in the field of sustainable agriculture.”

>>>>> Second done this in terms of use of the scale. This revision appears on Page 33, Line 18 to Page 34, Line 17 of the revised manuscript.

“This study's innovative approach, which maps psychological criteria onto farmers' Net Zero commitments, represents a significant advancement in agricultural climate policy. It integrates diverse criteria by combining elements of Net Zero Policy Commitment with well-cited psychological theories, including the Unified Theory of Acceptance and Use of Technology, the Theory of Planned Behavior, the Prototype-Willingness Model, Implementation Intentions, Literacy, Eudaimonia, and Self-Determination Theory. This holistic view goes beyond the traditional focus areas of policy and technology, delving into the psychological and behavioral aspects of farmers. The survey covers a comprehensive range of Net Zero commitment areas, from emission reduction targets to financial incentives and adaptation strategies, ensuring a deep understanding of farmers' perceptions and interactions with these aspects. Therefore, this model offers a nuanced yet parsimonious understanding of the intricate relationship between policy requirements and the psychological dimensions of farmers' behaviors and attitudes towards Net Zero commitments. This approach could lead to more effective strategies for implementing these commitments in the agricultural sector by considering how the practical and psychological aspects of sustainable farming transitions align. To illustrate the practical applications of our findings, the Integrated Motivation Model for Sustainable Farming scale could be utilized by policymakers to design interventions that closely align with farmers' psychological needs. For example, if the Integrated Motivation Model for Sustainable Farming reveals a strong influence of community norms on farmer behavior, policymakers could initiate community-led programs that leverage peer influences to promote sustainable practices. These programs might include peer mentoring systems where experienced farmers who have successfully adopted sustainable techniques share their practices and experiences. Such community-focused interventions can harness the collective power, enhancing the acceptance and integration of sustainable practices within local farming communities.”

3. **Moreover, Study 2's validation of the proposed psychological model through Confirmatory Factor Analysis and concurrent validity assessment represents a significant contribution to the field. However, considering the geographical variation in agricultural practices, particularly between the UK and the US, it would be informative to explore potential cross-cultural differences in the psychological determinants of Net Zero farming.**

>>>>> To address UK/USA we performed an additional analysis comparing scores on the new scale, presenting the statistics in a new table (Table 6). The text for this revision appears on Page 27, Line 12 to 25 of the revised manuscript.

“Finally, an independent groups t-test was conducted to compare the scores on each of the scales between farmers in the USA and the UK. The decision to use an independent t-test was supported by the skewness statistics for each group, which ranged from -0.85 to -0.358, indicating a normal distribution. Table 6 presents the mean scores, standard deviations, t-values, p-values, and effect sizes for these tests. Considering multiple comparisons were made across seven psychological scales, a Bonferroni correction was applied to adjust the significance level to p < 0.007. Using this stringent criterion, significant differences in the scores for 'Sustainable Farming Readiness and Confidence' and 'Sustainable Incentive Engagement and Acceptance' were found. In assessing the magnitude of these differences between USA and UK farmers, effect sizes were evaluated using Cohen’s d (49), with values of 0.2, 0.5, and 0.8 representing small, medium, and large effects, respectively." In terms of the effect sizes of these significant differences, the effect size for 'Sustainable Farming Readiness and Confidence’ was small, and the effect size for 'Sustainable Incentive Engagement and Acceptance' was medium.”

>>>>> We also provide a discussion of these findings on Page 32, Line 23 to Page 33, Line 17 of the revised manuscript.

“The findings from employing both UK and USA samples revealed significant differences in two of the subscales of the Integrated Motivation Model for Sustainable Farming scale: 'Sustainable Farming Readiness and Confidence' and 'Sustainable Incentive Engagement and Acceptance,' with farmers in the USA scoring significantly higher and demonstrating small and medium effect sizes, respectively. The 'Sustainable Farming Readiness and Confidence' factor encompasses aspects such as strategy integration, emission reduction targets, and a comprehensive understanding of Net Zero practices, reflecting farmers' readiness and capabilities in adapting to these practices. The higher scores among farmers in the USA could be attributed to well-established agricultural extension services in the United States (50), which may better equip farmers with the necessary literacy about Net Zero practices and the skills to implement detailed strategies and coping mechanisms. Similarly, the 'Sustainable Incentive Engagement and Acceptance' factor, which assesses attitudes toward utilizing financial incentives for Net Zero practices, also showed higher scores among farmers in the USA. This may be explained by the differences in agricultural policy frameworks between the two countries. In the United States, there is a robust system of financial incentives for sustainable farming under various federal and state programs, likely reflected in the higher engagement and acceptance scores. Meanwhile, the agricultural policy framework in the UK has been undergoing significant changes with new schemes currently being introduced following the Agriculture Act 2020 (51) and the exit from the European Union’s Common Agricultural Policy.”

4. **The passage effectively integrates a wide array of psychological frameworks to elucidate the complexities of farmers' adoption of sustainable practices. However, to enhance clarity and coherence, it could benefit from a more structured presentation of these frameworks, perhaps organized into distinct sections or subsections based on thematic similarities or theoretical foundations. This structural refinement would assist readers in navigating the diverse theoretical perspectives and their interconnections more seamlessly.**

>>>>> As discussed with point 1 of this reviewer, This consideration led to major changes in the structure and positioning of the paper, during which we realized that the psychological theories needed to be organized within a wider context. Therefore, we have separated the frameworks into broadly behavioral adoption—based on existing literature—and purpose-driven, which introduces more value-based considerations. By defining these, we believe the literature is more organized, and the paper makes a more significant contribution to the field. We also feel that this helps cement many aspects of the paper in terms of providing a wider context for the interpretation of the model's findings and describing the resulting model. This revision appears on Page 4, Line 10 to Page 6, Line 13 of the revised manuscript.

“In this consideration, we present two main psychological contexts that influence farmers' decision-making towards sustainable farming: Behavioral-Adoption and Purpose-Driven frameworks. The Behavioral-Adoption frameworks, often cited in sustainable farming literature, focus on external factors like social influence and practical utility, which are crucial for the adoption of new technologies and practices by farmers. For instance, these frameworks explore how peer attitudes and the availability of supportive conditions can encourage sustainable behaviors. On the other hand, the Purpose-Driven frameworks delve into the internal motivations of farmers, such as personal fulfillment and alignment with core values, which drive their engagement in sustainable practices. These frameworks emphasize understanding why farmers choose to engage in sustainability from an intrinsic perspective. By contrasting and integrating these frameworks, our study offers a comprehensive view of the dynamic interplay between external influences and internal motivations that shape sustainable agricultural practices..

In terms of how these two sets of frameworks relate to specific psychological model and sustainable farming, the first set to consider are Behavioral-Adoption frameworks. Am exampler model is the Unified Theory of Acceptance and Use of Technology (1), which explains technology acceptance by highlighting factors like performance expectancy (the technology’s perceived usefulness), social influence (the impact of peer attitudes), and facilitating conditions (the presence of a supportive environment). It is these elements of the Unified Theory of Acceptance and Use of Technology model that significantly influences farmers’ attitudes toward adopting sustainable farming practices (2). Complementing the Unified Theory of Acceptance and Use of Technology, the Theory of Planned Behavior (3) provides a framework around adopting sustainable technologies in farming via intentions, shaped by attitudes, norms, and perceived control, predict behaviors. Research shows that attitudes, social norms, and perceived control positively influence farmers' intentions to adopt practices like organic farming and improved natural grassland management (4–6).

The second set to consider are Purpose-Driven frameworks that focus on beliefs, meaning, fulfillment, desired prototypes, environmental literacy processes, and contributions to personal and community well-being. Within the existing sustainable farming literature, Self-Determination Theory (SDT;(7)) has been used to explore practices in sustainable farming as reflections of purpose-driven intentions in farming, in terms of motivational intrinsic versus extrinsic needs driven by autonomy, competence, and relatedness. Rsearch shows that SDT principles are instrumental in how a self-directed approach contributes to sustainable farming, emphasizing how intrinsic choices over external rewards and fostering conditions that support farmer autonomy leads to sustainable farming (8,9). Moreover, integrating models such as the Prototype Willingness Model (10) and the theory of Implementation Intentions (11,12) could offer an advanced understanding of concepts of prototypical influences around the identity of being a sustainable farmer (e.g., the ‘eco-friendly farmer’) and intentional, planned behaviors (e.g. specific plans) in sustainable farming are Purpose-Driven factors in farming. For example, in terms of implementation intentions, developing detailed action plans for pesticide reduction or water conservation can underline the practical applicability of these theories. (13,14). There are also other psychological framework that we consider Purpose-Driven. The inclusion of models of environmental literacy (15) that enriches our understanding by framing the competencies necessary for sustainable farming. This approach not only emphasises individuals' abilities to access and understand information relating to sustainable farming, but also emphasis the ability to make purposeful and well-informed decisions that positively impact on sustainable farming. Lastly, though there has been an exploration of the relationship between everyday affect (e.g. enjoyment) and farming practices (16,17) there is a compelling need for a broader examination of well-being concepts, particularly eudaimonia. Eudaimonia focuses on fulfilling one's potential and finding deep meaning and happiness in alignment with one’s true self (18,19). Exploring how eudaimonia correlates with sustainable farming practices (20) offers vital insights into possibly why fulfilment and meaning may be purposefully connected with the adoption of sustainable practices in farming.”

5. **Furthermore, while the passage highlights the importance of simplifying and unifying psychological frameworks for practical application, it could provide more concrete examples or case studies illustrating how this integration process could be operationalized in real-world contexts. By demonstrating the application of the proposed unified model in specific agricultural settings or initiatives, readers would gain a clearer understanding of its potential implications and utility for stakeholders in the field.**

>>>>> In terms of the new structure around the behavioral-adoption and purpose-driven frameworks, we can make this integration a lot clearer. Therefore, we have revised this paragraph to discuss not only how integration translates complexity into practical action in terms of parsimony but also how it bridges the gap between intricate psychological theories and the realities of farming. In this paragraph, we provide examples concerning water resources and climate change. This revision appears on Page 6, Line 14 to Page 7, Line 14 of the revised manuscript.

“These two broad psychological frameworks, Behavioral-Adoption and Purpose-Driven, support the psychological, motivational, cognitive, and social processes that influence farmers' decisions towards sustainable farming. Although the wealth of models (1,3,7) offers deep insights into the adoption of sustainable practices, their complexity can hinder the creation of straightforward, unified strategies. By identifying common themes across these frameworks, we can simplify complex theories into practical strategies that prevent siloed approaches (an approach where different sectors or disciplines operate in isolation) and enhance farmers' and land managers' adoption of sustainable practices. This integration helps bridge the gap between intricate psychological theories and farming realities, promoting a more cohesive understanding. For example, in regions where farming demands significant water resources, leading to environmental stress, especially during drought conditions, promoting water-efficient technologies poses a challenge (21). Farmers may be hesitant due to high upfront costs, uncertainty about effectiveness, and disruption of established practices. A better understanding can be achieved by integrating factors from Behavioral-Adoption and Purpose-Driven frameworks. We could identify factors that emphasize practical readiness and external factors such as financial incentives (Behavioral-Adoption), which align with farmers' values towards sustainability and stewardship of natural resources (Purpose-Driven). This integration provides a more meaningful and strategic understanding of these factors. Similarly, in areas where farming is crucial to the economy and vulnerable to climate change (22), a unified model that integrates Behavioral-Adoption strategies—demonstrating the effectiveness and efficiency of new sustainable practices—with the Purpose-Driven aspects that engage farmers' values and cultural practices related to land and community welfare, can be advantageous. This dual approach ensures farmers are not only prepared but also motivated to adopt new practices that benefit both their livelihood and their community. Thus, this unified approach addresses both the logistical and motivational aspects of adopting or changing behaviors to target sustainable farming.”

6. **Additionally, the passage could delve deeper into the potential challenges or limitations associated with integrating multiple psychological frameworks, such as conflicting theoretical assumptions or methodological differences. Acknowledging and addressing these challenges would add depth to the discussion and provide a more nuanced understanding of the complexities involved in developing a comprehensive psychological model for sustainable agriculture.**

>>>>> We have addressed this issue, and given the new structure and framework – this discussion, on both theory and methodological approaches has been useful in establishing a further case for the study. This revision appears on Page 7, Line 15 to Page 8, 10 of the revised manuscript.

“Integrating psychological frameworks, however, presents certain challenges. A primary concern is the potential conflict between theoretical assumptions. Behavioral-Adoption frameworks, for example, often emphasize external motivations like incentives or regulatory pressures, which may contradict the intrinsic motivations highlighted by Purpose-Driven frameworks. This conflict can create tension in designing interventions that need to balance immediate benefits with long-term sustainable motivations. Additionally, there are methodological challenges related to scale compatibility and data integration when merging the Behavioral-Adoption and Purpose-Driven frameworks. These frameworks not only originate from different theoretical backgrounds but also use distinct scales and measurement tools that may not directly align. For instance, some theories such as Eudaimonia **(19)** and Self-Determination Theory **(23)** feature psychometrically validated scales providing clear, measurable indicators. Others, like the Unified Theory of Acceptance and Use of Technology **(1)** and health literacy models **(24)**, require scales specifically adapted to the behavior under study. To address these issues, we propose adopting the abductive method (25)—a reasoning approach that begins with observations and seeks the simplest, most likely explanation. This leads us to suggest developing a new model that integrates diverse psychological factors, focusing specifically on sustainable farming within the NetZero context. This model would incorporate new constructs along with measures that capture the motivational, cognitive, and social processes influencing farmers. By synthesizing diverse models into a unified one, we aim to move beyond siloed approaches and create an integrated framework that effectively promotes sustainable practices in farming.”

7. **Finally, while the passage outlines the methodological approach for developing and validating the proposed psychological model, it could provide more detail on the specific steps involved in data collection, analysis, and interpretation. Providing transparency regarding the research methodology would enhance the credibility and replicability of the study, enabling readers to assess the rigor of the findings and their applicability to other contexts.**

>>>>> Apologies we left some details out of the paper, and they have been reinstated. First in more information on the recruitment of Sample 1 – that we mistakenly left out the use of Prolific – which we had referred to in Study 2, but not 1. This revision appears on Page 9, Line 22 to Page 10, Line 9 of the revised manuscript.

“Recruitment was conducted through the online crowdsourcing platform Prolific, utilizing two methods to reach potential participants. The first method targeted individuals in the UK who were registered on the site as working in the agricultural industry, identified through a screening question regarding employment in the 'Agriculture, Food, and Natural Resources' sector, ensuring a relevant participant pool. The second method invited individuals who self-identified as farmers to register for a short screening study. This was necessary because those on the crowd-sourcing site might not have originally registered their employment sector with the crowdsourcing platform due to reasons such as lack of perceived relevance, privacy concerns, changes in role, or misalignment with professional identity. Subsequently, those who were not already identified via the first method were invited to the main study via the crowdsourcing platform, thereby broadening the potential respondent base.”

>>>>> We have also provided more information on the interpretation of the factor analysis. This revision appears on Page 16, Lines 10 to 16 of the revised manuscript.

“The factor interpretation, conducted by the study's author, focused on the relationship between the psychological factors identified and their practical implications for sustainable farming. Each of the seven factors derived from the exploratory factor analysis offers a relatively unique view, being identified as separate factors within the EFA, on the motivations and barriers farmers face in adopting sustainable practices. The interpretation, which relied on the written content of items, helped align the theoretical frameworks with real-world applications.”

8. **The discussion effectively outlines the seven-factor model and the development of the ANZAR Scale, providing valuable insights into the psychological dimensions of farmers' engagement with Net Zero practices. However, to enhance clarity and coherence,** **it could benefit from a more structured presentation, perhaps organizing the discussion around each factor or scale development process individually. This would help readers navigate the complex interplay of psychological theories and empirical findings more seamlessly, facilitating a deeper understanding of the research outcomes.**

>>>>> Due to the restructuring of the paper around behavior adoption and purpose-driven approaches, this structure has informed other aspects. Therefore, in terms of making sense of the 7-factor model, we have integrated and described this model in terms of the dynamics. We would argue that this suggestion by the reviewers has strengthened the paper—and this conceptualization is now introduced in the second paragraph of the discussion. This revison appears on Page 30, Line 24 to Page 31, Line 24 of the revised manuscript.

“We introduce the Integrated Motivation Model for Sustainable Farming, which defines seven key factors by merging two distinct frameworks presented in this paper: Behavioral-Adoption and Purpose-Driven. The Behavioral-Adoption framework is focused on enhancing practical readiness, skill development, and strategic implementation necessary for adopting sustainable practices. It emphasizes external motivations such as incentives, compliance with standards, and new technology utilization. The five factors identified in the study—Sustainable Farming Readiness and Confidence, Sustainable Incentive Engagement and Acceptance, Climate Adaptation Competence and Confidence in Agriculture, Net Zero Accountability and Reporting Commitment, and Innovation and Technological Competence in Net Zero Farming—represent this framework. They underscore the importance of equipping farmers with the necessary tools and information, creating environments conducive to sustainable practices, and ensuring farmers feel competent and supported. Conversely, the Purpose-Driven framework delves into intrinsic and value-based motivations driving sustainable farming. It aligns farmers’ actions with their personal values and community norms, fostering deeper engagement and commitment to sustainability. Factors such as Sustainable Agricultural Commitment and Stewardship, and Community Influence and Commitment in Sustainable Farming reflect this framework, focusing on internal satisfaction and personal fulfillment driven by a responsibility towards the community and environment. The interplay of these frameworks within our model offers a dual perspective that captures the motivational, cognitive, and social processes influencing sustainable farming. This integration helps us understand not only the practical steps necessary for behavioral adoption but also the deeper sense of purpose sustaining these changes over time. By illuminating the complex interplay between Behavioral-Adoption and Purpose-Driven factors, our model provides a holistic view of what drives farmers towards achieving Net Zero goals. It becomes a crucial tool for researchers, policymakers, and practitioners in the field of sustainable agriculture.”

9. **Additionally, while the discussion highlights the practical implications of the findings for policymakers and practitioners, it could provide more specific examples or case studies illustrating how the ANZAR Scale could be utilized in real-world contexts. Concrete examples demonstrating how the scale could inform the design of targeted interventions or support mechanisms tailored to farmers' psychological profiles would enhance the applicability of the research findings and offer actionable insights for stakeholders in the agricultural sector. Furthermore, the discussion acknowledges the limitations of the study, such as its cross-sectional nature and the need for future research to explore longitudinal designs and regional policy environments.** **While these limitations are appropriately addressed, providing suggestions for how future studies could mitigate or overcome these limitations would add depth to the discussion and guide researchers in designing more robust investigations in the future.**

>>>>> In response to your constructive comments, we have enhanced the discussion section by providing specific examples of how the Scale can be utilized to design interventions and support mechanisms, particularly focusing on the construct of 'Community Influence and Commitment'. These examples aim to offer actionable insights that are directly applicable to stakeholders within the agricultural sector. This revision appears on Page 34, Lines 5 to 17 of the revised manuscript.

“This approach could lead to more effective strategies for implementing these commitments in the agricultural sector by considering how the practical and psychological aspects of sustainable farming transitions align. To illustrate the practical applications of our findings, the Integrated Motivation Model for Sustainable Farming scale could be utilized by policymakers to design interventions that closely align with farmers' psychological needs. For example, if the Integrated Motivation Model for Sustainable Farming reveals a strong influence of community norms on farmer behavior, policymakers could initiate community-led programs that leverage peer influences to promote sustainable practices. These programs might include peer mentoring systems where experienced farmers who have successfully adopted sustainable techniques share their practices and experiences. Such community-focused interventions can harness the collective power, enhancing the acceptance and integration of sustainable practices within local farming communities.”

>>>>>. Additionally, we have detailed suggestions for future research to address the current study's limitations. These suggestions aim to guide researchers in designing more robust investigations, particularly through longitudinal studies and the examination of regional policy influences, thereby enriching the depth and utility of future research in this domain. This revision appears on Page 34, Line 18 to Page 35, Line 18 of the revised manuscript.

“However, it is important to acknowledge the limitations of the study, including: 1) its broad nature, 2) its cross-sectional design, and 3) its focus on psychological mechanisms. First, given the broad scope of the study, exploring the role of regional farming and policy environments would provide valuable context to our findings. Examining the impact of regional policy environments in a more granular manner could reveal how specific policies influence psychological outcomes and sustainable behaviors in diverse farming contexts. Future studies could then offer more customized and effective recommendations for policy adjustments and support mechanisms tailored to regional needs. Second, regarding the cross-sectional design, future research could employ longitudinal designs to track the evolution of farmers' attitudes and behaviors over time. This approach would allow researchers to better ascertain causality and the durability of psychological factors influencing sustainable farming practices. Third, concerning the focus on psychological mechanisms, although the work has established integrated models across various psychological contexts, advocating for interdisciplinary collaboration is crucial to further enhance our understanding of sustainable agriculture. The integration of insights from psychology, agronomy, economics, and environmental science not only enriches our approach but is essential in tackling the multifaceted challenges associated with achieving Net Zero emissions in agriculture. For instance, while agronomic innovations provide the tools for sustainable farming, psychological insights into farmer behavior and economic analyses of market and policy impacts are equally critical for the adoption and effectiveness of these technologies. Environmental science connects these elements to the broader ecological outcomes, ensuring that sustainable agricultural practices align with global environmental goals. Together, integrating these disciplines contributes to a comprehensive strategy that addresses both the practical and theoretical aspects of sustainability, creating a resilient agricultural framework that can adapt and thrive in the face of climate challenges. To some extent, the current findings demonstrate the potential for adopting this interdisciplinary approach.”

10. **Finally, the discussion could emphasize the importance of interdisciplinary collaboration in advancing research on sustainable agriculture.** **Highlighting the potential benefits of integrating insights from disciplines such as psychology, agronomy, economics, and environmental science could underscore the need for a holistic approach to addressing the complex challenges of achieving Net Zero emissions in agriculture.**

>>>>> We have expanded the discussion to highlight the importance of interdisciplinary collaboration in the field of sustainable agriculture. We now emphasize the essential roles of psychology, agronomy, economics, and environmental science in developing a holistic approach to achieve Net Zero emissions. This revision underlines our commitment to integrating diverse academic disciplines, thereby enriching the theoretical foundation and practical implications of our research in sustainable agriculture. This revision appears on Page 35, Lines 4 to 18 of the revised manuscript.

“Third, concerning the focus on psychological mechanisms, although the work has established integrated models across various psychological contexts, advocating for interdisciplinary collaboration is crucial to further enhance our understanding of sustainable agriculture. The integration of insights from psychology, agronomy, economics, and environmental science not only enriches our approach but is essential in tackling the multifaceted challenges associated with achieving Net Zero emissions in agriculture. For instance, while agronomic innovations provide the tools for sustainable farming, psychological insights into farmer behavior and economic analyses of market and policy impacts are equally critical for the adoption and effectiveness of these technologies. Environmental science connects these elements to the broader ecological outcomes, ensuring that sustainable agricultural practices align with global environmental goals. Together, integrating these disciplines contributes to a comprehensive strategy that addresses both the practical and theoretical aspects of sustainability, creating a resilient agricultural framework that can adapt and thrive in the face of climate challenges. To some extent, the current findings demonstrate the potential for adopting this interdisciplinary approach.”
